# The cost-effectiveness of a family meetings intervention to prevent depression and anxiety in family caregivers of patients with dementia: a randomized trial

**DOI:** 10.1186/1745-6215-14-305

**Published:** 2013-09-22

**Authors:** Karlijn J Joling, Judith E Bosmans, Harm WJ van Marwijk, Henriëtte E van der Horst, Philip Scheltens, Janet L MacNeil Vroomen, Hein PJ van Hout

**Affiliations:** 1 Department of General Practice and Elderly Care Medicine, VU University Medical Centre, EMGO + Institute for Health and Health Care Research, Van der Boechorststraat 7, 1081, BT Amsterdam, The Netherlands; 2 Faculty of Earth and Life Sciences, VU University Amsterdam, De Boelelaan 1085, 1081, HV Amsterdam, The Netherlands; 3 Department of Neurology, VUmc Alzheimer Center, De Boelelaan 1118, 1081, HZ Amsterdam, The Netherlands; 4 Department of Internal Medicine, Section Geriatrics, Amsterdam Medical Center, Meibergdreef 9, 1105, AZ Amsterdam, The Netherlands

**Keywords:** Caregivers, Cost-effectiveness, Dementia, Family meetings, Informal care

## Abstract

**Background:**

Dementia imposes a heavy burden on health and social care systems as well as on family caregivers who provide a substantial portion of the care. Interventions that effectively support caregivers may prevent or delay patient institutionalization and hence be cost-effective. However, evidence about the cost-effectiveness of such interventions is scarce. The aim of this study was to evaluate the cost-effectiveness of a family meetings intervention for family caregivers of dementia patients in comparison with usual care over a period of 12 months.

**Methods:**

The economic evaluation was conducted from a societal perspective alongside a randomized trial of 192 primary caregivers with community-dwelling dementia patients. Outcome measures included the Quality Adjusted Life-Years (QALY) of caregivers and patients and the incidence of depression and anxiety disorders in caregivers. Missing cost and effect data were imputed using multiple imputations. Bootstrapping was used to estimate uncertainty around the cost-differences and the incremental cost-effectiveness ratio (ICER). The bootstrapped cost-effect pairs were plotted on a cost-effectiveness plane and used to estimate cost-effectiveness curves.

**Results:**

No significant differences in costs and effects between the groups were found. At 12 months, total costs per patient and primary caregiver dyad were substantial: €77,832 for the intervention group and €75,201 for the usual care group (adjusted mean difference per dyad €4,149, 95% CI -13,371 to 21,956, ICER 157,534). The main cost driver was informal care (66% of total costs), followed by patients’ day treatment and costs of hospital and long-term care facility admissions (23%). Based on the cost-effectiveness acceptability curves, the maximum probability that the intervention was considered cost-effective in comparison with usual care reached 0.4 for the outcome QALY per patient-caregiver dyad and 0.6 for the caregivers’ incidence of depression and/or anxiety disorders regardless of the willingness to pay.

**Conclusions:**

The annual costs of caring for a person with dementia were substantial with informal care being by far the largest contributor to the total societal costs. Based on this study, family meetings cannot be considered a cost-effective intervention strategy in comparison with usual care.

**Trial registration:**

ISRCTN register, ISRCTN90163486

## Background

Dementia is a common and disabling disorder in elderly people and the number of people affected is expected to rise exponentially [1]. The majority of people with dementia live in the community where informal caregivers are the main providers of care. Providing care for a relative with dementia can be stressful and demanding, leading to a high burden and significant physical and mental health problems [2-7] compared to non-caregivers [8]. The worldwide costs of dementia (US$604 billion in 2010) already amount to more than 1% of the global gross domestic product [9]. Further, dementia patients have increased healthcare utilization rates compared with other major diseases [10-12]. Previous research showed that informal care costs make up a substantial part of the total annual costs of dementia. In the United States, the care provided by informal caregivers to people with dementia was valued at more than $202 billion in 2010 [13]. Also, in the United Kingdom and Scandinavia, informal care account for a substantial proportion (55% and 33%, respectively) of the annual cost of dementia [14,15]. In several countries, the current health care workforce may not be numerous enough to meet the care needs of the increasing number of older patients; this might place even more pressure on family and other informal caregivers in the near future [16-19].

Dementia thus imposes a heavy economic burden on the social care system, as well as on family and friends who provide unpaid care. Interventions which effectively support caregivers and prevent psychiatric morbidity may postpone institutionalization of the patient with dementia [20,21], decrease work absenteeism and healthcare utilization of the caregiver and may hence be cost-effective in comparison with usual care. Information about the trade-off between costs and benefits of caregiver interventions is urgently needed since it will enable policy makers to understand the magnitude of the economic consequences and to decide whether it is efficient to implement such interventions considering the scarce resources available for healthcare. A large number of studies evaluated the effectiveness of supportive interventions for informal caregivers of people with dementia [22-24], but recent reviews showed that there are only few economic evaluations of such interventions. Moreover, the available studies in this field often have insufficient methodological quality and measured costs from a narrow perspective [25,26]. Some studies analysed effect and cost differences between groups separately instead of comparing the total cost of each option against the total effects. Furthermore, often not all relevant cost categories such as informal care costs and lost productivity costs were taken into consideration, and hence, these studies were not performed from a societal perspective. The systematic review of Jones et al. [25] on the cost-effectiveness of interventions targeted at informal dementia caregivers identified only four psychosocial interventions and four other types of non-pharmacological interventions that were evaluated in studies with sufficient quality [25]. Only one of these psychosocial interventions reported to be cost-effective compared with usual care [27].

A psychosocial intervention targeted at the whole family, such as family meetings, may be a potentially cost-effective approach. Family meetings may maximize the positive contribution of family members, decrease caregiver burden, alleviate psychological symptoms [28,29] or even prevent the primary caregiver from developing a psychiatric disorder, such as a depressive or anxiety disorder. This might enable the caregiver to provide care for a longer time, thereby leading to decreased healthcare utilization and loss of productivity and postponement of patient institutionalization. Previously, a multi-component intervention program for caregivers that consisted of four family meetings, two individual counselling sessions with the caregiver, support group participation and ad hoc counselling was shown to decrease depressive symptoms in informal caregivers and to result in a substantial delay in time until nursing home placement of patients [20,21,28-30]. Joling et al. subsequently investigated, in a pragmatic trial, whether a structured family meetings intervention could prevent mental disorders and reduce the severity of symptoms in caregivers of persons with dementia thus delaying institutionalization of patients. Clinically, the effects were smaller than anticipated. Although the incidence of depression and anxiety disorders was substantial in the sample of caregivers, the intervention did not prevent the onset of disorders, nor reduce symptom levels or delayed time until institutionalization of patients compared to usual care [31-33]. Evidence on the cost-effectiveness of family meetings is still lacking; little is known about the costs associated with such interventions and the relationship between costs and effects. This article presents the first cost-effectiveness analysis alongside a randomized trial of a family meeting intervention for caregivers of patients with dementia in comparison with usual care.

## Methods

### Study design, setting and participants

The economic evaluation was performed alongside a randomized trial to evaluate a family meetings intervention in comparison with usual care from a societal perspective. The follow-up of the study was 12 months. Caregiver and patient dyads were recruited through memory clinics (n = 91), organizations delivering case management (n = 79), general practices, home care settings and meeting centres for people with dementia and their caregivers (n = 22) in the Netherlands. Caregivers were eligible if they were the primary family caregiver of a community-dwelling relative with a clinical diagnosis of dementia and had at least one other family member or friend available to participate in the family meetings. If there was more than one family caregiver caring for the patient, the primary caregiver was defined as the person who coordinated the caring process, usually the person who spent most hours on caregiving tasks. Caregivers were excluded when 1) they met the criteria for a clinical depressive or anxiety disorder as measured with the Mini International Neuropsychiatric Interview (MINI) [34], 2) their relative with dementia was scheduled to move into a nursing home, 3) they presented with severe somatic or psychiatric co-morbidity which would significantly impair cooperation with the study. Persons who gave written informed consent and had sufficient command of the Dutch language were eligible for participation in the study; the design of the study has been described in detail elsewhere [31]. The Medical Ethics Committee of the VU University Medical Centre approved the study protocol.

### Randomization and blinding

After obtaining signed informed consent and baseline measurements, dyads of patients and their primary family caregiver were randomized by an independent researcher stratified by recruitment centre in blocks of four to either usual care or the family meetings intervention. The interviewers who measured the outcomes were blinded to randomization status. Blinding of participants and the counsellors conducting the family meetings was not possible due to the nature of the intervention.

### Intervention

The family meetings intervention has been described in detail elsewhere [31,32]. Briefly, caregivers randomized to the intervention group were invited to participate in six in-person counselling sessions: one individual preparation session, followed by four structured meetings that included their relatives and/or friends (family meetings), and one additional individual evaluation session. The family meetings were held once every 2 to 3 months in the year following enrolment in the program. The aim of the family meetings was to offer psycho-education, teach problem-solving techniques and mobilize the existing family networks of the patient and primary caregiver in order to improve emotional and instrumental support. The content of the sessions was guided by the needs of the caregiver. The intervention protocol recommended that patients would not attend the family meetings, unless the caregiver strongly desired the patient to be present. Ad hoc telephone counselling from the same counsellor was available to caregivers and their families beyond the scheduled sessions. The counsellors who led the family meetings had an advanced degree in nursing, social work, psychology or an allied profession and were trained prior to the study by the research team. The total estimated time for the intervention was 6.5 hours per patient-caregiver dyad, including the time spent for the individual and family sessions (5.5 hours) and administration and preparation time for the counsellor (1 hour). Intervention participants also had access to all the usual types of care.

### Usual care

Participants randomized to the usual care group were free to use all types of care, including community-based mental health services or support resources other than family meetings at any time throughout the 12 months follow-up, reflecting standard care. Usual care in the Netherlands may consist of a range of health and social care services and can differ across participants. However, family meetings are rarely organized or offered in a structured way with follow-up sessions. They also tend to focus on providing clinical information and not on increasing family support and relieving the caregiver.

### Effect outcomes

Effect outcomes were the quality of life of the patient and caregiver (as a dyad and separately) and the incidence of major depressive or anxiety disorders in caregivers. Quality-of-life of caregivers and patients was measured using the SF-12 at baseline and at 6 and 12 months [35]; the caregiver rated the patient’s quality of life. The tariff developed by Brazier et al. [36] was used to convert health states to utilities [36]. Utilities express the relative desirability of a health state on a scale of 0 ('death’) to 1 ('perfect health’). QALYs were calculated by multiplying the utilities with the amount of time a person spent in a particular health state. Transitions between health states were linearly interpolated.

The incidence of depression and anxiety was measured at baseline and at 3, 6, 9 and 12 months after enrolment with the MINI [34], a short diagnostic interview for DSM-IV mental disorders that can be used for psychiatric evaluation and outcome tracking.

### Cost outcomes

Cost data were collected from a societal perspective using two consecutive cost diaries that covered a period of 6 months each as well as 6-monthly interview assessments to measure informal care time; direct and indirect costs of both the caregiver and patient were gathered. Table 1 lists the cost categories and prices used in this economic evaluation. All costs were adjusted to the year 2009 using consumer price indices if necessary. The year 2009 was chosen because most cost data were collected during that year. Costs were calculated by multiplying the units of resource use by their cost price according to the Dutch guidelines for health economic evaluations [37]. If no standard cost was available, tariffs were used. Medication costs were valued using prices of the Royal Dutch Society for Pharmacy [38]. Lost productivity costs were calculated according to the friction cost approach (friction period 154 days) using the mean age and sex specific income of the Dutch population [39,40]. The intervention costs were calculated based on the time investment of the counsellors including 15 minutes of administration by the counsellors per family session and the time spent by the counsellors on the individual and family counselling sessions (€45.35 per hour). Travel costs and capital costs were not included. Informal caregiver time was valued using a shadow price based on the hourly cost of a legally employed cleaning person (€12.50 per hour) as recommended in the Dutch guidelines. Caregivers were asked how much time they spent on a list of informal care tasks during the previous day. Informal care tasks included: support with activities of daily living (ADL), support with instrumental activities of daily living (IADL), household activities of daily living (HDL) and supervision tasks. The total informal care time was calculated by adding up the reported time on these tasks during the previous day, using a maximum of 24 hours. We extrapolated the time per day reported at baseline, 6 and 12 months to determine the total amount of carer time during 12 months of follow-up.

**Table 1:** Prices used in the economic evaluation and utilization of health care resources and work absenteeism for caregivers and patients with complete cost data during the 12-month follow-up period

**Cost category**	**Unit**	**Unit cost**	**Caregivers**	**Patients**
		**(€, 2009)**	**(n = 125)**	**(n = 118)**
			**Mean (SD)**	**Mean (SD)**
Ambulatory care (except home care)				
General practitioner	Contact	28^a^	5.0 (4.3)	4.9 (5.4)
Paramedical therapist	Contact	Variable^b^	7.3 (14.3)	10.2 (19.9)
Psychologist or psychotherapist	Contact	80 or 77	0.5 (2.7)	0.2 (0.7)
Social worker	Contact	65	0.2 (1.0)	0.4 (2.0)
Social psychiatric nurse	Contact	45	1.7 (3.3)	1.8 (3.6)
Psychiatrist	Contact	171	0.03 (0.4)	0.2 (0.7)
Other counsellor^c^	Contact	Variable	0.3 (1.5)	n/a
Peer support group counselling	Session	68	1.8 (3.8)	0.1 (0.7)
Outpatient appointment	Contact	72	2.6 (3.0)	3.9 (3.8)
Home care and other support				
Domestic home help	Hour	24	10.6 (17.5)	see caregiver
Professional home care	Hour	44	0.5 (4.2)	5.4 (15.8)
Respite care	Hour	12.50	1.6 (8.3)	n/a
Meal supply at home	Meal	6	2.7 (20.6)	3.4 (22.8)
Day treatment and admissions				
Day treatment	Day	251	1.6 (8.2)	48.3 (67.2)
Admission, elderly home	Day	90	0.2 (2.3)	1.1 (7.6)
Admission, nursing home	Day	238	0.4 (3.8)	15.2 (46.1)
Admission, academic hospital	Day	575	0.2 (2.0)	0.3 (1.8)
Admission, non-academic hospital	Day	435	0.4 (1.9)	1.0 (5.5)
Admission, intensive care unit	Day	2183	0.02 (0.2)	none
Absenteeism				
Paid labour	Day	Friction costs^d^	2.2 (11.7)	n/a
Unpaid labour	Hour	12.50	4.7 (20.7)	n/a
Informal care	Hour	12.50	see patient	3760.3 (3273.0)

^a^Prices of GP telephone contacts and GP home visits involved €14 and €43, respectively.

^b^Paramedical therapists included: physiotherapist, manual therapist, Cesar exercise therapists, alternative therapists. For each profession, the appropriate guideline price was used and if not available, the mean of the price according to five therapists was used.

^c^Other counsellors included counselling sessions with a caregiver consultant, a coordinator of meeting centres for persons with dementia and their caregivers or a practice nurse. Prices were according to the professional organization.

^d^Costs for paid labour were calculated on the basis of a mean income of the Dutch population according to age and sex.

### Power calculation

The power calculation was based on the expected effects of the intervention on the main outcome measure, incidence of a depression or anxiety disorder. The yearly incidence of disorders among caregivers at risk was estimated at 30% [3]. The trial was powered to detect a 20% decrease in the incidence. We calculated that 73 participants per group would be needed, assuming a 2-sided test, an alpha of 0.05 and a power of 80%. With a dropout of 20%, at least 182 participants were needed.

### Statistical analysis

The economic evaluation included a cost-utility analysis with the QALY as the effect (analysed for the patient-caregiver dyad as well as for caregivers and patients QALYs separately) and a cost-effectiveness analysis with the caregiver’s incident depression and/or anxiety disorder as the effect. The statistical analyses were performed according to the intention-to-treat principle (ITT). Multiple imputation was used to impute missing cost and effect data. Variables found to be related to cost and effect outcomes and missing follow-up data, were included in the multiple imputation model. Each of the 10 imputed data sets were separately analysed and the results of the 10 analyses were pooled using Rubin’s rules [41]. To adjust for selection bias, variables with significant baseline differences between the intervention and usual care group (caregiver anxiety score (HADS-A) [42], age of the patient and age of the caregiver) were incorporated as covariates in the analyses. For costs, linear regression models were estimated. Costs generally have a highly skewed distribution; therefore, bootstrapping with 5,000 replications was used to estimate bias-corrected and accelerated confidence intervals around cost differences [43,44]. Cost-effectiveness was expressed as an incremental cost effectiveness ratio (ICER) for the intervention compared to the control condition, a measure of the additional cost per unit of health gain. ICERs were calculated by dividing the difference in total costs between the intervention and usual care group by the difference in clinical effects. A bivariate regression model was estimated with separate regression equations for costs and effects including covariates. Non-parametric bootstrapping was also used to estimate the uncertainty surrounding the incremental cost-effectiveness and cost-utility ratios (5,000 replications). The bootstrapped cost-effect pairs were plotted on a cost-effectiveness plane and used to estimate cost-effectiveness acceptability (CEA) curves. In a cost-effectiveness plane, incremental costs between the intervention and usual care are plotted on the y-axis and incremental effects on the x-axis resulting in four quadrants. The northeast quadrant indicates that the intervention is more expensive and more effective than usual care. In the southeast quadrant the intervention dominates usual care, i.e. is less expensive and more effective than usual care. In the southwest quadrant the intervention is less expensive and less effective than usual care. Finally, in the northwest quadrant the intervention is dominated by usual care (more expensive and less effective). Most newly developed interventions are more expensive and more effective than usual care, which implies that a trade-off needs to be made about whether the additional benefits justify the additional costs. This decision depends on the societal willingness to pay for an additional unit of effect. However, this willingness to pay is generally not known. CEA curves show the probability that the intervention is cost-effective in comparison with the control treatment for a range of willingness to pay values [45]. Finally, two sensitivity analyses were carried out. Complete case analyses were performed using only persons with complete follow-up cost and effect data to assess whether missing data might have caused bias. Furthermore, the ITT analyses were repeated without adjustment to assess the impact of the baseline imbalances and to check the robustness of the results.

## Results

### Participant flow and recruitment

**Figure 1:**
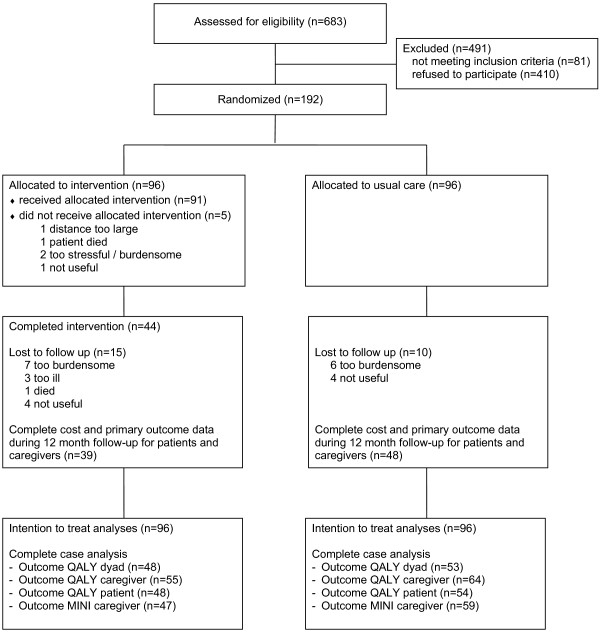
Flow chart of the study sample.

Participants were recruited from November 2007 to November 2009. Figure 1 presents the flow chart of the study sample. Of the caregivers assessed for eligibility, 192 met all inclusion criteria and were willing to participate. Reasons for exclusion included not meeting the inclusion criteria (n = 81) and refusal of participation (n = 410) and were described in detail elsewhere [32]. Summarized, the primary reason for refusal was a claimed lack of need for this intervention. There were no significant differences between the patient-caregiver dyads that refused participation and the participating dyads in gender, caregiver-patient relation and the type of service they were recruited from.

### Baseline characteristics

Table 2 presents the socio-demographic and clinical characteristics of the caregivers and patients at baseline. Patients and caregivers in the intervention group were significantly younger (patient’s age: t = 3.07, degrees of freedom (df) = 188.10, 95% CI for difference in means: 1.37 to 6.33, and caregivers’ age: t = 2.27, df = 188.47, 95% CI: 0.45 to 6.30) and caregivers in the intervention group had higher levels of anxious symptoms (HADS-A score) (t = -2.51, df = 187, 95% CI for difference in means: -2.22 to -0.27) at baseline than participants in the usual care group.

**Table 2:** Baseline demographic and clinical characteristics of the caregivers and patients

	**Caregiver**		**Patient**	
	**Intervention (n = 96)**	**Usual care (n = 96)**	**Intervention (n = 96)**	**Usual care (n = 96)**
Age, M (SD)	67.8 (9.8)*	71.2 (10.7)	72.8 (9.1)*	76.7 (8.3)
Female gender, n (%)	67 (69.8)	68 (70.8)	30 (31.3)	32 (33.3)
Spouse of the patient, n (%)	92 (95.8)	89 (92.7)		
Living with patient, n (%)	93 (96.9)	91 (94.8)		
Educational level, n (%)				
Elementary/Lower	28 (29.2)	34 (35.4)	42 (43.8)	44 (45.8)
Secondary	37 (38.5)	30 (31.3)	30 (31.3)	28 (29.2)
Higher/ University	29 (30.2)	32 (33.3)	24 (25.0)	22 (22.9)
Utility score SF6D, M (SD)	0.8 (0.01)	0.7 (0.01)	0.7 (0.01)	0.7 (0.01)
Anxiety score HADS-A (0–21), M (SD)	6.1 (3.4)*	4.8 (3.5)		
Depression score CES-D (0–60), M (SD)	12.1 (7.9)	10.8 (7.1)		
ADL independencies (out of 6), M (SD)			5.1 (1.4)	5.3 (1.1)
IADL independencies (out of 7), M (SD)			2.7 (1.8)	2.6 (1.5)
MMSE (0–30), M (SD)			21.4 (4.9)	21.7 (5.6)

M: Mean; SD: Standard deviation; HADS-A: Hospital Anxiety and Depression Scales- Anxiety subscale; CES-D: Centre for Epidemiologic Studies Depression Scale; (I) ADL: (Instrumental) activities of daily living; MMSE: Mini Mental State Examination.

*significant difference with usual care group (*P* <0.05).

### Numbers analysed

Complete follow-up data (cost data and both the QALY and MINI effect outcome data) were available for 44 (46%) intervention and 57 (59%) usual care group caregivers. For patients, complete follow-up data were available for 48 patients (50%) in the intervention group and 54 patients (56%) in the usual care group. Missing follow-up data was significantly associated with the number of ADL dependencies (mean difference 0.44, 95% CI 0.09 to 0.79), the number of IADL dependencies (mean difference 0.75, 95% CI 0.29 to 1.21) and lower mini mental state examination scores (mean difference -1.92, 95% CI -3.46 to -0.37) of patients at baseline. In accordance with the ITT principle, missing follow-up data were imputed and all participants were included in the analyses.

### Uptake of the intervention

Of those randomized to the intervention group, 91/96 participated in the preparation session, 73/96 attended 1 or 2 family meetings and 44/96 adhered (i.e., completed the preparation session plus 3 or 4 family meetings within 12 months) to the intervention protocol.

### Costs

Table 3 presents the mean costs and adjusted differences in costs for caregivers and patients between the intervention and usual care group. Ambulatory care costs in the intervention group were significantly lower. Further, considerable investments need to be done to provide the intervention in addition to usual care. Total costs were €77,832 per patient-caregiver dyad in the intervention group and €75,201 in the usual care group (mean adjusted difference €4,149, 95% CI -13371; 21956). Patient costs were the majority of the total costs (€73,854 in the intervention group and €70,684 in the control group per patient) and only a relatively small amount concerned costs for caregivers (€3,979 in the intervention group and €4,517 in the control group per caregiver). Although statistically non-significant, costs for caregivers were in favour of the intervention group (mean adjusted difference €788, 95% CI -3529; 1439), while for patients costs were lower in the usual care group (mean adjusted difference €4,936, 95% CI -11808; 21750), which was mainly due to lower informal care costs. Informal care costs were by far the largest contributor to total dyadic costs (66%) with an average amount of €50,859. This means that caregivers spent on average 11 hours per day on caregiving, including supervision. Another large cost driver involved day treatment and admissions of the patient to hospitals and long-term care facilities which amounted to an average of €17,756 per patient. The volumes of resource use for patients and caregivers with complete cost data are shown in Table 1.

**Table 3:** Unadjusted and adjusted differences in costs (€) for caregivers and patients after 12 months of follow-up

**Cost category**	**Intervention (n = 96)**	**Usual care (n = 96)**	**Unadjusted difference**	95% CI^*^	Adjusted difference^**^	95% CI^*^
	**M (SD)**	**M (SD)**				
**Caregiver**						
Ambulatory care	842 (103)	1,110 (124)	-268	-571; 20	-335	-663; -28^***^
Day treatment and admissions	854 (454)	1,074 (436)	-220	-1371; 1081	-107	-1327; 1272
Home care and other support	751 (171)	1,253 (247)	-502	-1151; 7	-438	-1015; 57
Absenteeism	1,133 (640)	691 (458)	442	-742; 2058	57	-1366; 1436
Medication	271 (48)	389 (57)	-118	-266; 19	-94	-259; 48
Intervention	129 (10)	0	129	110; 147^***^	129	109; 149^***^
Total caregiver	3,979 (884)	4,517 (876)	-538	-2976; 1681	-788	-3529; 1439
**Patient**						
Ambulatory care	1,057 (115)	1,016 (126)	41	-274; 328	-81	-438; 221
Day treatment and admissions	18,388 (2639)	17,124 (2735)	1264	-6122; 8436	1208	-6017; 8620
Home care and other support	1,451 (425)	1,581 (482)	-130	-1362; 1121	-109	-1439; 1177
Medication	1,097 (131)	1,106 (102)	-9	-284; 308	-98	-374; 182
Informal care	51,860 (4585)	49,858 (4065)	2002	-10082; 13877	4017	-8541; 16715
Total patient	73,854 (6106)	70,684 (5706)	3170	-13398; 19776	4936	-11808; 21750
**Total patient- caregiver dyad**	77,832 (6384)	75,201 (5997)	2631	-14520; 20118	4149	-13371; 21956

^*^95% CI obtained by bias corrected and accelerated bootstrapping.

^**^Cost differences adjusted for variables that differed significantly between intervention and usual care group at baseline (age caregiver, age patient, HADS anxiety score).

^***^Indicates a statistically significant difference between the intervention and usual care group.

### Effects

Full details on the clinical outcomes were presented in the accompanying clinical paper [32,33]. Briefly, a substantial number (37/96; 39.6%) of caregivers in the intervention group and in the usual care group (34/96; 35.4%) developed a depressive or anxiety disorder within 12 months. The incidence was similar in both groups (adjusted difference 0.01; 95% CI -0.14 to 0.17, Table 4). There was no statistically significant difference in quality of life for either the patients, caregivers or patient-caregiver dyads (Table 4).

**Table 4:** Results of the cost-effectiveness analyses for the outcomes MINI and QALYs

**Analysis**	**Outcome**	**N**	**∆ effects**	**95% CI**	**∆ costs**	**95% CI**^**#**^	**ICER**	**Distribution cost-effectiveness plane (%)**			
								**NE**	**SE**	**SW**	**NW**
ITT Unadjusted	Patient-caregiver dyad QALY	192	0.006	-0.05; 0.06	2631	-14520; 201118	438299	31	27	12	30
	Caregiver QALY	192	0.004	-0.04; 0.04	-538	-2976; 1681	-149984	15	43	25	17
	Patient QALY	192	0.002	-0.04; 0.04	3169	-13398; 19776	1313110	31	25	11	33
	Caregiver incidence of depression and/or anxiety (MINI)*	192	-0.04	-0.20; 0.11	-538	-2976; 1681	12604	8	21	47	24
ITT Adjusted	Patient-caregiver dyad QALY	192	0.04	-0.03; 0.08	4149	-13483; 21965	157534	54	30	3	13
	Caregiver QALY	192	0.02	-0.005; 0.05	-788	-3551; 1453	-32254	24	71	3	2
	Patient QALY	192	0.002	-0.03; 0.04	4936	-11631; 22073	2574938	35	19	9	37
	Caregiver incidence of depression and/or anxiety (MINI)*	192	0.01	-0.14; 0.17	-788	-3566; 1399	-59011	14	44	31	12
CCA	Patient-caregiver dyad QALY	101	-0.005	-0.08; 0.07	3951	-17662; 25955	-807703	23	20	16	40
	Caregiver QALY	119	0.02	-0.02; 0.05	-483	-2514; 1720	-24472	25	62	6	8
	Patient QALY	102	-0.02	-0.07; 0.03	4373	-16422; 25402	-240247	12	12	22	54
	Caregiver incidence of depression and/or anxiety (MINI)*	106	0.08	-0.06; 0.21	-711	-2783; 1775	-9271	20	68	9	4

ITT: Intention to treat; CCA: Complete case analysis.

^**#**^95% CI obtained by bias corrected and accelerated bootstrapping.

*An effect difference >0 means that over a period of 12 months the risk of an incident depression and/or anxiety disorder was lower in the intervention group compared to the usual care group.

### Cost-effectiveness

**Figure 2:**
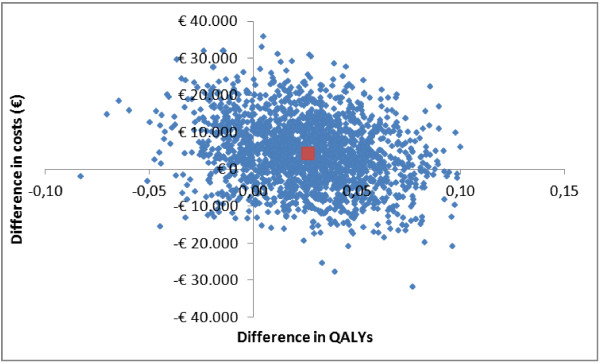
Cost-effectiveness plane for the difference in Quality Adjusted Life-Years (QALYs) per dyad gained at 12 months.

**Figure 3:**
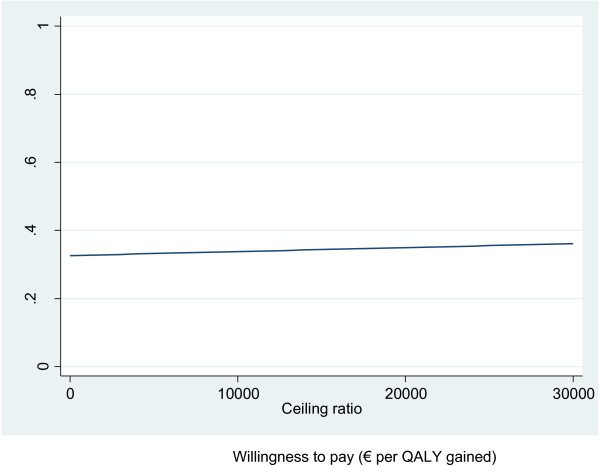
**Cost-effectiveness acceptability curve for the outcome Quality Adjusted Life-Years (QALYs) per dyad.** Willingness to pay (€ per QALY gained).

Since the differences in effects on all outcomes were very small, this resulted in very large ICERs that will be very sensitive to uncertainty in incremental effect (Table 4). Figure 2 shows that most cost-effect pairs for the outcome QALY per care dyad (patient and caregiver combined) were contained in the northeast quadrant of the cost-effectiveness plane indicating some higher effects accompanied by some higher costs of the intervention compared with usual care (non-significant). The cost-effectiveness analysis for the QALY was repeated for patients and caregivers separately, but this revealed no significant differences either (Table 4). Based on the CEA curves, the probability that the intervention was considered cost-effective in comparison with usual care was 0.33 for the outcome QALY per care dyad when the ceiling ratio is set at €0/QALY and was 0.36 for a ceiling ratio of €30,000/QALY (Figure 3). For caregivers separately, this probability was 0.73 for a ceiling ratio of €0/QALY and 0.85 for a ceiling ratio of €30,000/QALY. For patients, the probability remained around 0.29 for both ceiling ratios of €0/QALY and €30,000/QALY. For infinite values of willingness to pay, the maximum probabilities were 0.74, 0.95 and 0.54 for the dyads, caregivers and patients, respectively (figures for caregivers and patients separately not shown).

**Figure 4:**
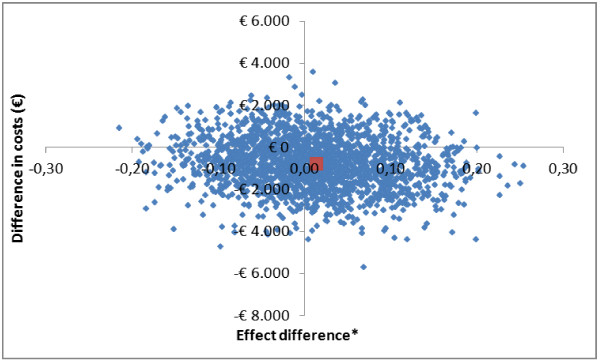
**Cost-effectiveness plane for the incidence of depression and/or anxiety disorders in caregivers.** *An effect difference >0 means that over a period of 12 months the risk of an incident depression and/or anxiety disorder was lower in the intervention group compared to the usual care group.

Figure 4 shows the cost-effectiveness plane for the outcome incident depression and/or anxiety, which incorporated the cost and effects for the caregiver only. The majority of the cost-effectiveness pairs (75%) were located in the southern quadrants, with a somewhat higher proportion in the Eastern quadrant, suggesting that the intervention has lower costs accompanied by higher effects for caregivers compared with usual care, although these differences were statistically non-significant. The CEA curve for this outcome shows that the maximum probability that the intervention is cost-effective in comparison with usual care was 0.73 at a willingness to pay of zero euros. With increasing values for willingness to pay, the probability that the intervention is cost-effective in comparison with usual care goes towards 0.57. The curve decreases because the cost difference for caregivers is in favour of the intervention group (Figure not shown).

### Sensitivity analyses

Two sensitivity analyses were carried out. The first involved a complete case analysis using only persons with complete follow-up data. For complete cases, the intervention was not considered cost-effective in comparison with usual care on any of the outcome measures. Effect differences for all outcomes remained small and non-significant (Table 4). The total costs per care dyad (patient and caregiver combined) with complete data were €64,732 (SE 55,869) for the intervention group and €63,303 (SE 48,982) for the usual care group (mean adjusted cost difference €3,951, 95% CI -17,662; 25,955). The second sensitivity analysis contained a repetition of the ITT analysis without adjustment for baseline imbalances between the intervention and control group. Also for this analysis the intervention was not considered cost-effective in comparison with usual care on any of the outcome measures and effect and cost differences for all outcomes remained non-significant (Table 4). Compared to the adjusted ITT analysis, only the MINI outcome reversed in favour of the usual care group, but this still involved a small and non-significant difference.

## Discussion

### Main findings and interpretation

This study is one of the first to investigate the impact of a dementia caregiver intervention from a societal perspective. Total annual costs were substantial and amounted to an average of €77,832 and €75,201 per care dyad (patient and caregiver combined) in the intervention and usual care group, respectively. These costs were much larger than expected based on previous estimates of dementia care costs [9,46]. Besides, almost half of the caregivers developed a mental disorder [32]. Moreover, this is the first study investigating whether a structured family meetings intervention for caregivers of persons with dementia is cost-effective in comparison with usual care. The most important cost drivers involved informal care costs and day treatment and admission costs of hospital and long-term care facility admissions of the patient. Over 12 months, we observed no significant differences in total costs between both groups. Based on the differences per cost category, our hypotheses that this intervention may decrease work absenteeism and healthcare utilization of the caregiver were not confirmed. Only ambulatory care costs differed amongst carers in the intervention and usual care group and were, unexpectedly, found to be increased amongst intervention carers. It could be possible that the intervention might have had an opposite effect by making caregivers realise they were stressed and therefore needing to seek help, increasing their use of ambulatory care. There were also no differences between groups in QALYs for both patients and caregivers or on clinical mental parameters for caregivers. Although, total costs for caregivers in the intervention group were somewhat lower than in the usual care group and effects were somewhat larger, cost-effectiveness planes showed that there was substantial uncertainty. Based on these findings, we conclude that family meetings are not cost-effective in comparison with usual care. A possible explanation for the non-significant findings was the minimal contrast between the intervention and control group as a consequence of the high level of standard care in the Netherlands. Future studies should focus on caregiving profiling based on who is most in need of family meetings support and who would benefit the most from this type of intervention. Besides, research should indicate to what extent or how it is possible to increase access, uptake and adherence.

### Comparison with the existing literature

Our findings are in line with recent systematic reviews that demonstrated little evidence for the cost-effectiveness of non-pharmacological interventions for dementia patients and their informal caregivers [25,26]. Since this is the first cost-effectiveness study on family meetings, comparison of our results with similar studies is difficult. Overall, there is only little evidence on the cost-effectiveness of interventions supporting informal caregivers. From the systematic review of Jones et al. [25] only two randomized controlled trials can be identified that evaluated the cost-effectiveness of psychosocial interventions targeting informal caregivers of dementia patients that were performed from a societal perspective, which is considered as the most appropriate for economic evaluations [47]. The study by Graff et al. [27] found that community occupational therapy for dementia patients, including a training programme for caregivers in coping behaviours and supervision, was cost-effective in comparison with usual care, and specifically reduced costs of informal caregiving [27]. Mean costs per patient involved €12,563 in the intervention group and €14,311 in the usual care group after 3 months of follow-up and were thus lower than in our study. Cost of caregivers’ healthcare utilization and medication were not reported. The results of another economic evaluation by Wilson et al. [48] were in line with our findings. In this cost-utility study on a structured befriending service among 236 carers of people with a primary progressive dementia, the intervention was not found to be cost-effective [48]. Mean QALYs per carer over 15 months were only slightly higher (0.017) in the intervention group compared with the control groups and mean costs from a societal perspective were £1,813 higher. The randomized trial by Roberts et al. [49] that was included in the systematic review of Jones [25] evaluated the effects of an individualized problem-solving counselling intervention for caregivers and the expenditures of their health care utilization, but costs and effects were analysed separately [49]. Hence, this study cannot be considered a cost-effectiveness study.

Previously, the World Alzheimer Report [9] described that in high-income countries, the direct costs of social care (professional care in the community, and the costs of residential and nursing home care) amounted the most to the costs of dementia care (nearly 50%) [9]. In our study, day treatment and admissions were also an important cost driver, but informal care costs accounted for the majority of the total costs (65%). This is far higher than estimations in other cost of dementia studies. In the World Alzheimer Report [9], costs of informal care contributed 42% to total costs worldwide [9]. This can partly be explained by the fact that, in this report, informal care included only tasks associated with basic ADL and IADL, while in our study supervision time was also incorporated. In the present study, informal caregivers were mainly spouses living together with the person with dementia and part of them reported 'being on duty’ 24 hours a day. Further, all patients in our sample were living in the community at baseline, while the World Alzheimer Report evaluation also consisted of people with dementia living in residential or nursing home care facilities. Other costing studies were more in line with our estimate and reported high informal care costs. Wilson et al. [48] found that costs of informal care contributed 85% to the total costs on a befriending program for carers of people with dementia [48]. In the study of Graff et al. [27] investigating occupational therapy for dementia patients [27], caregivers provided, on average, 11 hours a day which is similar with our findings. These numbers emphasize the importance of informal caregiving for people with dementia living in the community.

An interesting question is why so many of our sample developed a depressive or anxiety disorder. Caregivers were mainly women caring for their spouse with dementia. These groups of caregivers appear to be more vulnerable to experience adverse psychosocial and physical health effects [3,50,51]. Further, caregivers who experience high burden and have psychological complaints may have been more willing to participate in a trial testing a psychosocial intervention and therefore it is possible that recruited caregivers were at a higher risk of developing depression. On the other hand, the fact that they were willing to receive support may also have reduced their risk. The incidence in the present study is far higher than found in 'general’ elderly cohorts [52-54] and is in line with the estimate of 48% reported in the review of Cuijpers et al. [3] on depressive disorders in caregivers of dementia patients [3]. This is an alarming finding and stresses the importance of further efforts to improve support for these caregivers. It is already known that caregiving for dementia patients is more stressful than caregiving for other older people, because of the specific demands that dementia poses on the caregiver such as being 'on duty’ 24 hours a day and the fact that it often lasts much longer than other caregiving, which might explain the high vulnerability of this group [8,55].

### Generalizability

Although a substantial number of caregivers refused to participate in our study, patients and caregivers in our study did not differ significantly from the persons who declined participation with regard to gender, patient-caregiver relation and the service they were recruited from. Therefore, broadly, our sample seems to be representative for the population of caregivers and patients that receives care. The pragmatic character of this study, with few *a priori* exclusion criteria, resembling usual circumstances as much as possible greatly enhances the generalizability of our results to the population of caregivers and patients receiving care in daily practice.

### Strengths and limitations

The major strength of this study is the detailed coverage of all relevant costs in this specific population, including detailed measurement of informal care. In this way, we were able to estimate the impact of the intervention from a societal perspective. The substantial contribution of informal care costs indicates the importance of including these costs when performing an economic evaluation in the field of dementia. Other strengths of this study involve the randomized design, the pragmatic approach that increases the applicability of the results to daily practice, and the relatively large sample size and long follow-up period. A limitation of the study is the rate of incomplete data. Complete follow-up data of costs and both the QALY and MINI effect outcomes were available for 101/192 (53%) caregivers and 102/192 (53%) patients. Data on costs were self-reported by the caregiver using cost diaries, which was rather time consuming for caregivers. Caregivers who considered withdrawal were offered a minimal assessment that included only the main effect parameters. This minimized dropout during the study, but decreased the completeness of cost data. On the other hand, the rate of incomplete data is partly due to the multi-factorial nature of data collection (especially on the resource use side), and is consistent with that seen in other studies. Poorer patients’ health was significantly associated with having incomplete follow-up data, which could have influenced the results. However, to minimize the impact, we used multiple imputations to impute missing data. Previous studies have shown that simple procedures to handle missing data, like complete case analysis, can bias the cost estimates considerably and therefore multiple imputation is recommended to account for missing data [56,57]. In addition, results of the complete case analyses were in line with the main analyses, which strengthen the conclusion of this study that the intervention was not cost-effective in comparison with usual care.

## Conclusions

This family meetings intervention for caregivers of persons with dementia was not cost-effective compared with usual care. Cost-effectiveness planes showed that there was considerable uncertainty. The limited contrast between the intervention and control group because of the high level of standard care in the Netherlands could have decreased the cost-effectiveness of the intervention. Future research should focus on factors that determine whether caregivers take up family meetings, and whether or how it is possible to increase the uptake and adherence, which might improve effectiveness and cost-effectiveness of the intervention. Although our study did not show any significant effects of family meetings or cost savings, the substantial number of caregivers who developed a mental disorder and the high informal care costs in both groups emphasize the need to search for interventions that support caregivers of dementia patients cost-effectively across the course of the illness.

## Abbreviations

ADL: Activities of daily living; CEA: Cost-effectiveness acceptability; HDL: Household activities of daily living; IADL: Instrumental activities of daily living; ICER: Incremental cost-effectiveness ratio; ITT: Intention-to-treat; MINI: Mini International Neuropsychiatric Interview; QALY: Quality Adjusted Life-Years.

## Competing interests

Funding from this study was obtained from the Netherlands Organization for Health Research and Development (ZonMw), grant number 62300040. The funders had no role in study design, data collection and analysis, decision to publish, or preparation of the manuscript. The authors declare that they have no competing interests.

## Authors’ contributions

KJ managed the trial, performed the statistical analysis and drafted the manuscript. JB assisted with the data analysis and helped to draft the manuscript. HPH acted as principal investigator. KJ, HPH, HM, HEH, PS and JB participated in the design of the study. JMV checked the cleaning and transformation of the data. All authors contributed to writing the manuscript, read and approved the final manuscript.
